# Synergistic Effects of Supplemental Irrigation and Foliar Selenium Application on Dynamics Characteristics of Soil Respiration and Its Components in Millet Field

**DOI:** 10.3390/plants15060984

**Published:** 2026-03-23

**Authors:** Xiaoli Gao, Xuan Yang, Binbin Cheng, Haowen Wang, Yamin Jia

**Affiliations:** 1 College of Water Resources Science and Engineering, Taiyuan University of Technology, Taiyuan 030024, China; gaoxiaoli01@tyut.edu.cn (X.G.); yangxuan125800@gmail.com (X.Y.); chengbb2025@163.com (B.C.); 2 Shanxi Key Laboratory of Collaborative Utilization of River Basin Water Resources, Taiyuan 030024, China; 3 College of Water Mechanical and Vehicle Engineering, Taiyuan University of Technology, Taiyuan 030024, China; programcat123@gmail.com

**Keywords:** millet, supplemental irrigation, foliar selenium application, soil respiration, autotrophic respiration, heterotrophic respiration

## Abstract

Soil respiration (Rs) plays a pivotal role in carbon cycling within semi-arid ecosystems. In our millet field experiment, we measured Rs, autotrophic respiration (Ra), heterotrophic respiration (Rh), water consumption (ET), yield (Y), water use efficiency (WUE), and key soil environmental properties to examine the effects of supplemental irrigation and selenium application on Rs dynamics and to clarify the controlling factors. The experiment was conducted from 2023 to 2024 with four treatments and three replicates per treatment each year. These treatments comprised conventional rainfed (CK), supplemental irrigation (SI, 50 mm), rainfed with Se addition (CS, 67.84 g·hm^−2^), and supplemental irrigation with Se addition (SIS). SI increased CO_2_ emissions in the millet field, whereas selenium application (CS) suppressed them. Ra was the dominant component of Rs and was 1.03–4.01 times greater than Rh. SI and CS significantly affected cumulative CO_2_ emissions through Ra (*p* < 0.05), whereas their effects on Rh were minor. The CS treatment resulted in the lowest cumulative CO_2_ emissions at 4233 and 4009 g·m^−2^ in 2023 and 2024, respectively. Diurnal variation patterns of Rs, Ra, and Rh differed across millet growth stages. Both supplemental irrigation and selenium application improved soil water retention, soil enzyme activity, and soil organic matter (SOM), and moderated soil temperature. Classification and Regression Tree (CART) algorithm analysis revealed that Ra was primarily driven by soil temperature, with a feature weight of 86.95% determined by CART based on machine learning, whereas Rh was mainly influenced by soil enzyme activity, with a feature weight of 76.11%. The CS treatment enhanced production while promoting emission mitigation. The combined SIS treatment achieved the highest WUE and maintained a lower Rs than SI. These findings suggest an environmentally sustainable management strategy for millet production in semi-arid regions. However, due to the limited number of parcels in this study, further field-scale validation and additional experimental research involving multiple levels of supplemental irrigation and Se addition are necessary.

## 1. Introduction

Millet is generally recognized as a climate-resilient crop due to its strong drought tolerance and high nutritional value, particularly its rich selenium (Se) content, dietary fiber, essential amino acids, and phenolic compounds [1,2,3]. However, inadequate nutrient supply and limited rainfall restrict millet yields relative to major cereals, a challenge expected to intensify under future climate change [4,5]. Irrigation and fertilization remain the most fundamental and effective agronomic practices for improving crop productivity across agricultural systems [6]. These practices can significantly alter soil environmental conditions, including soil moisture, temperature, structure, crop physiological status, and enzyme activity, which subsequently affect greenhouse gas emissions in agroecosystems [7,8,9]. Understanding the dynamics of soil respiration (Rs) in millet farmland and its components is essential for clarifying soil carbon cycling processes and designing appropriate field management strategies.

Rs, comprising autotrophic respiration (Ra, root-driven) and heterotrophic respiration (Rh, microbial-driven), plays a key role in carbon cycling within semi-arid agroecosystems [10]. Millet shows different respiration patterns compared to other cereal systems due to its drought-adaptive physiology. Rs in the winter wheat phase was significantly lower than in the millet phase [11]. Gao reported that supplementary irrigation resulted in higher Rs and carbon emission fluxes than rainfed treatment, coinciding with greater yield and water use efficiency (WUE) [12]. Yang observed that biochar increased Rs by 33.3–63.5% compared with the control [13]. Soil cumulative carbon respiration increased by 13.37%, 21.55%, and 27.59% under nitrogen additions of 44.39, 88.77, and 133.16 μg·N·g^−1^ soil, respectively, showing a linear response to nitrogen input [14]. Li et al. showed that no-till increased Rs with increasing soil temperature and moisture, whereas straw mulch decreased soil moisture but tended to increase annual carbon emissions; straw incorporation resulted in the highest annual soil carbon emissions [15]. Most existing studies have examined millet Rs under single-factor conditions, such as irrigation, biochar addition, nitrogen application, or tillage. However, the synergistic effects of multiple management practices in millet systems remain insufficiently understood.

Water and fertilizer management regulate Rs through changes in soil physicochemical properties and microbial activity, including soil moisture, temperature, pH, redox potential (Eh), dissolved organic carbon (DOC), and microbial abundance and diversity [16,17,18]. Irrigation and fertilization also interact to influence Rs [19], and excessive water and nitrogen inputs can substantially increase greenhouse gas emissions [20]. Wang reported that Rs increased with soil moisture until an optimum threshold and then decreased when water content exceeded that threshold [21]. Wang et al. showed that reducing nitrogen input and improving water management techniques could decrease greenhouse gas emissions from grain production by 17% [22]. Yan found that nitrogen application promoted root growth and enhanced Rs in wheat at the jointing stage, but excessive nitrogen did not further increase Rs; nitrogen fertilization stimulated both Rh and Ra, although a threshold response to application rate was evident [23]. Zhang demonstrated that combining nitrogen with biochar and straw in maize systems increased Rs by 16.07–30.32% [24]. Yang noted that integrating water-saving irrigation with organic fertilizer improved irrigation WUE and reduced CO_2_ emissions in paddy systems [25]. These findings collectively indicate that the mechanisms regulating Rs under different management practices are complex.

Most research on the combined effects of irrigation and fertilization on Rs has focused on rice, maize, and wheat, particularly in the context of nitrogen and organic amendments. Se fertilization in millet systems has been shown to increase yield and grain Se content and provides an economical and safe means of improving Se intake for the general population [26]. Se addition also alters soil nutrients and microbial communities, which are closely associated with Rs variation [27,28]. However, the specific mechanisms supporting the effects of Se application on Rs in millet agroecosystems remain poorly understood. More importantly, there is a lack of information on how supplemental irrigation modulates the effects of Se application on Rs. The potential synergistic effects of supplemental irrigation and Se application on Rs, as well as on associated environmental parameters, remain largely uncharacterized.

Evidence indicates that appropriate supplemental irrigation during the jointing stage in normal or dry years ensures high WUE [29]. Foliar Se application at the heading stage is readily absorbed and transported from leaves to grains [30,31]. Based on these findings, we hypothesize that (1) supplemental irrigation at the jointing stage will significantly increase Rs by promoting root and microbial activity, and (2) foliar Se application at the heading stage will modify Rs through changes in soil nutrient availability and microbial community structure. Furthermore, we hypothesize that irrigation and foliar Se application will lead to synergistic effects on Rs and related environmental parameters. Accordingly, this study examines the combined mechanisms shaping Rs responses to supplemental irrigation at the jointing stage and Se application at the heading stage in millet fields. The analysis focuses on variation in Rs and its components, together with associated soil environmental factors and crop physiological parameters.

## 2. Results

### 2.1. Rs Rate and CO_2_ Emission in Millet Growth Period

#### 2.1.1. Rs Rate

**Figure 1:**
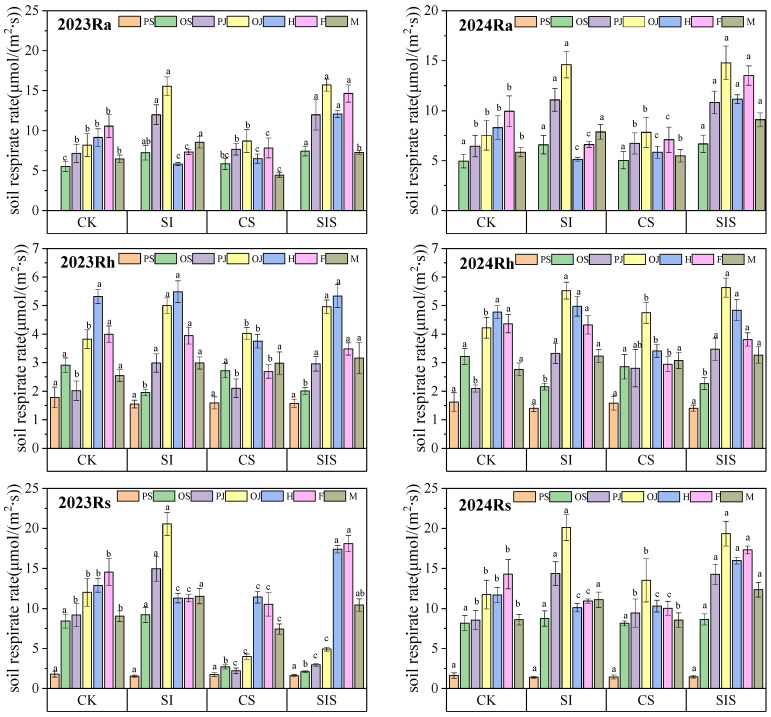
Variation in Rs rate and its components during the millet growth period under different treatments in 2023 and 2024. Error bars represent one standard deviation of three replicates. Different letters above bars indicate significant differences at *p* ≤ 0.05. PS, OS, PJ, OJ, H, F, and M represent pre-seedling, post-seedling, pre-jointing, post-jointing, heading, filling, and maturity stages, respectively.

As shown in Figure 1, Rs and its components followed a pattern of increasing and then decreasing across the millet growth period under all treatments in both years. Ra was 1.03–3.33 times higher than Rh in 2023 and 1.06–4.01 times higher in 2024, indicating that Ra is the dominant component in Rs. After supplemental irrigation at the pre-jointing stage, Rs and Rh under the SI treatment were significantly higher (*p* < 0.05) than those under CK. Following Se application at the heading stage, the CS treatment significantly reduced (*p* < 0.05) Rs and its components from heading to filling. During this period, Rs and Ra under SIS were significantly higher (*p* < 0.05) than the other treatments. Rh after the heading stage under SIS did not differ significantly (*p* > 0.05) from SI. Supplemental irrigation increased Rh during the post-jointing stage, whereas Se application reduced Rs and its components after the heading stage. Supplemental irrigation and foliar Se application showed a synergistic effect on Rs in millet fields.

#### 2.1.2. CO_2_ Emission

**Figure 2:**
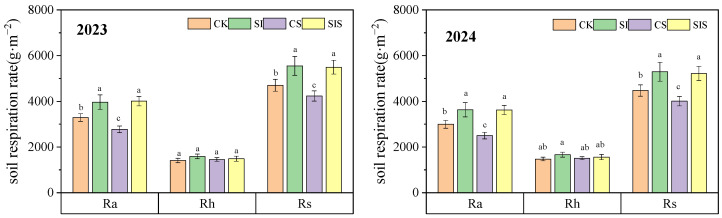
Cumulative CO_2_ emissions in millet fields under different treatments. Data are presented as mean ± standard deviation (SD) from three replicates per treatment. Different letters in the columns indicate significant differences among supplemental irrigation and Se application treatments at *p* < 0.05 according to the LSD test.

Cumulative CO_2_ emissions under SI were significantly higher (*p* < 0.05) than those under CK. By contrast, emissions under CS were slightly but significantly lower (*p* < 0.05) than those under CK. As shown in Figure 2, Ra contributed more to cumulative CO_2_ emissions than Rh across all treatments. Ra-derived emissions were 1.69–2.33 times higher than Rh-derived emissions. Supplemental irrigation combined with Se application demonstrated synergistic effects on cumulative CO_2_ emissions. Relative to CK, SI increased cumulative emissions by 18.12% and 18.40% (*p* < 0.05) in 2023 and 2024, whereas CS reduced emissions by 10.02% and 10.43% (*p* < 0.05), respectively. Under SIS, emissions were 16.84% and 16.77% higher than CK in the two years, with no significant difference (*p* > 0.05) from SI. The CS treatment produced the lowest cumulative CO_2_ emissions (4233 ± 223 and 4009 ± 206 g·m^−2^ in 2023 and 2024, respectively). Supplemental irrigation and Se application significantly influenced (*p* < 0.05) cumulative CO_2_ emissions through Ra, whereas their effects on Rh-derived emissions were minor. Compared with CK, SI increased Ra-derived emissions by 20.38% and 21.13% (*p* < 0.05), while CS reduced Ra-derived emissions by 15.65% and 16.66% (*p* < 0.05). Under SIS, Ra-derived emissions were 21.75% and 20.89% higher than CK in the respective years. The CS treatment recorded the lowest Ra-derived emissions (2777 ± 145 and 2499 ± 142 g·m^−2^ in 2023 and 2024, respectively). Overall, supplemental irrigation stimulated CO_2_ emissions, whereas Se application suppressed them.

### 2.2. Diurnal Variation in Rs Rate

**Figure 3:**
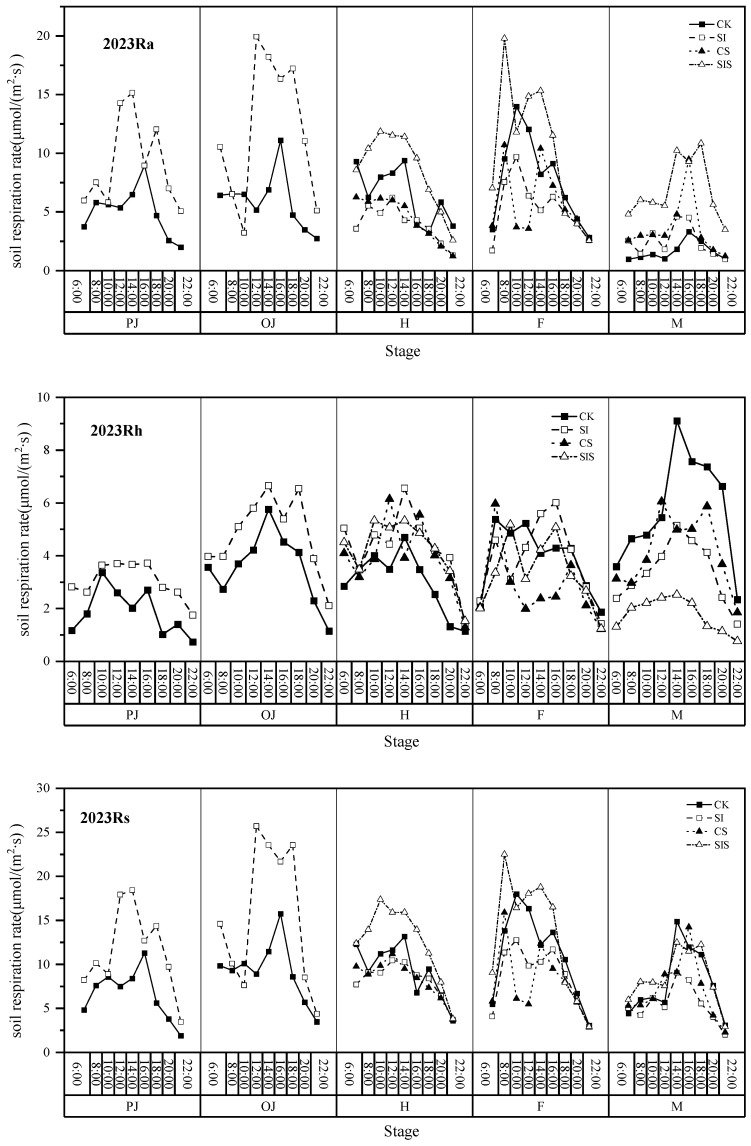
Diurnal variation patterns of CO_2_ respiration rates and components across growth stages in 2023.

**Figure 4:**
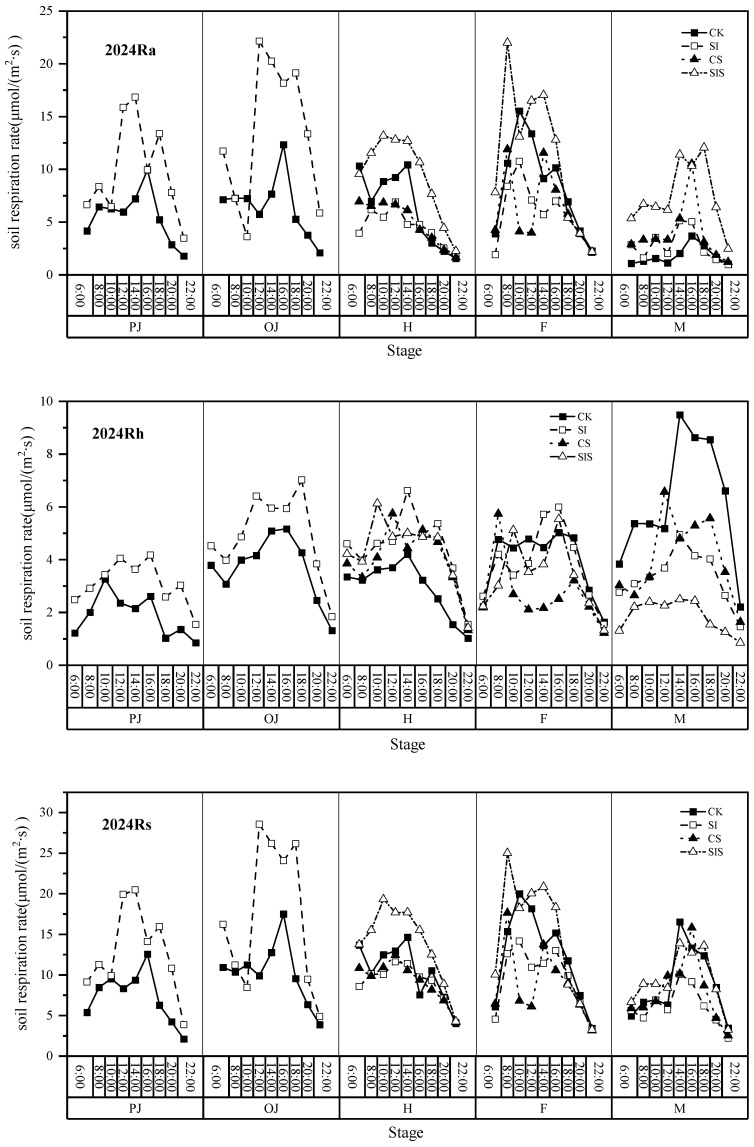
Diurnal variation patterns of CO_2_ respiration rates and components across growth stages in 2024.

Figure 3 and Figure 4 showed that, accounting for all of its components, Rs is generally higher during the day than the night, except at the maturity stage. Across growth stages, the most notable treatment effects emerged during heading and grain filling. At the heading stage, diurnal Rs and Ra generally followed the order SIS > CK > CS/SI, while Rh was consistently lowest in CK throughout the day. During the grain filling stage, SIS maintained higher Ra than other treatments, whereas Rh under CS was significantly lower than SI, SIS, and CK after 10:00. At maturity, as diurnal rhythms reversed, SIS continued to exhibit elevated Ra but reduced Rh relative to CK. The SIS treatment optimized diurnal respiratory dynamics by promoting Ra during heading and filling stages while suppressing Rh.

### 2.3. Water Consumption, Yield and WUE

Table 1 showed that supplemental irrigation and Se application significantly increased (*p* < 0.05) both grain yield and WUE. Relative to CK, SI and CS increased yield by 15.46% and 28.53% in 2023 and by 15.85% and 28.02% in 2024, respectively. Similarly, SI and CS increased WUE by 6.78% and 11.72% in 2023 and by 7.77% and 12.47% in 2024, respectively. Se fertilization further enhanced yield and WUE relative to SI. The combined SIS treatment produced the highest yield and WUE, increasing them by 43.36% and 23.69% in 2023 and by 43.38% and 25.19% in 2024. Se fertilization had a stronger effect (*p* < 0.05) on promoting yield than supplemental irrigation, and their combination resulted in an even greater increase in grain production.

### 2.4. Dry Matter Accumulation

**Figure 5:**
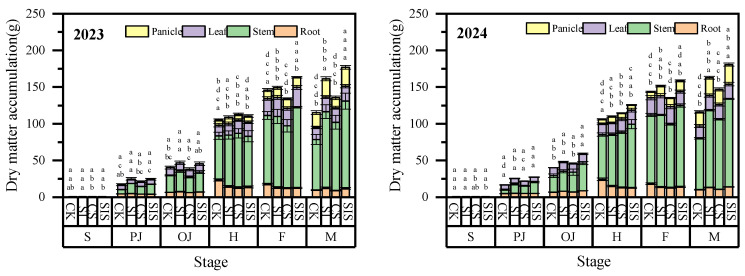
Variation in dry matter accumulation and distribution of millet under different treatments. Data are presented as mean ± standard deviation (SD) from three replicates per treatment. Different letters in the columns indicate significant differences among supplemental irrigation and Se application treatments at *p* < 0.05 according to the LSD test.

As shown in Figure 5, root and leaf dry matter increased and then decreased with crop development, peaking at the heading and filling stages, respectively. Stem dry matter increased continuously throughout the growth period. Panicle dry matter increased after heading and rose sharply during grain filling. During the filling stage, SIS treatment significantly reduced root dry matter (*p* < 0.05) but increased panicle dry matter compared with CK (*p* < 0.05). SI significantly increased stem dry matter during the post-jointing stage (*p* < 0.05), while its effect on leaf dry matter was not significant (*p* > 0.05). At heading, both SI and CS individually increased stem dry matter (*p* < 0.05). The SIS treatment caused a highly significant increase in both stem and panicle dry matter (*p* < 0.05). SIS treatment presented a synergistic effect in enhancing the transition from vegetative to reproductive growth, thereby contributing to increased grain yield.

### 2.5. Soil Environment

(1) Soil Moisture

**Figure 6:**
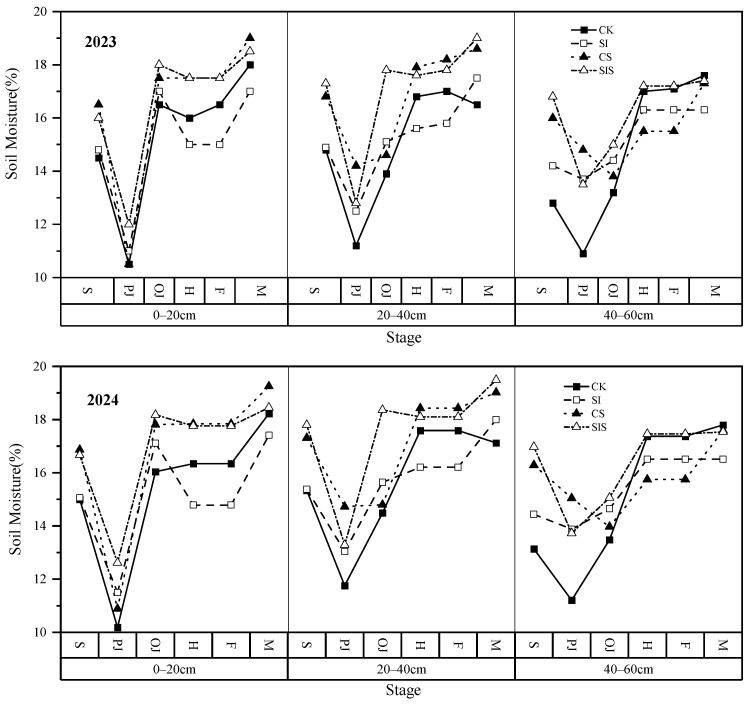
Soil moisture dynamics at different depths under each treatment.

Figure 6 shows soil moisture profiles in 2023 and 2024. In the absence of rainfall during the early jointing stage, soil moisture declined across all layers in every treatment. After supplemental irrigation in the late jointing stage, SI increased soil moisture relative to CK by 2.29–7.36%, 7.94–15.41%, and 7.95–8.56% in 2023 and by 2.10–6.65%, 7.97–24.09%, and 7.85–8.63% in 2024 at depths of 0–20 cm, 20–40 cm, and 40–60 cm, respectively. During the heading stage, with occasional rainfall, Se-treated plots maintained relatively stable soil moisture. In 2023, CS increased soil water content by 5.99–10.08% (0–20 cm), 4.73–11.55% (20–40 cm), and 2.47–9.12% (40–60 cm). In 2024, soil water content under CS increased by 5.63–9.20% (0–20 cm) and 4.78–11.12% (20–40 cm), while the 40–60 cm layer decreased by 3.53–9.32%. SIS significantly increased soil moisture relative to CK in both years. In 2023, increases were 6.44–20.74%, 8.70–11.90%, and 6.53–6.99% across the three soil layers. In 2024, increases were 6.01–20.07%, 8.29–11.66%, and 5.76–6.27%. Supplemental irrigation increased soil moisture, while Se fertilization improved water retention in the 0–40 cm profile. The combined SIS treatment enhanced water retention across all depths.

(2) Soil Temperature

**Figure 7:**
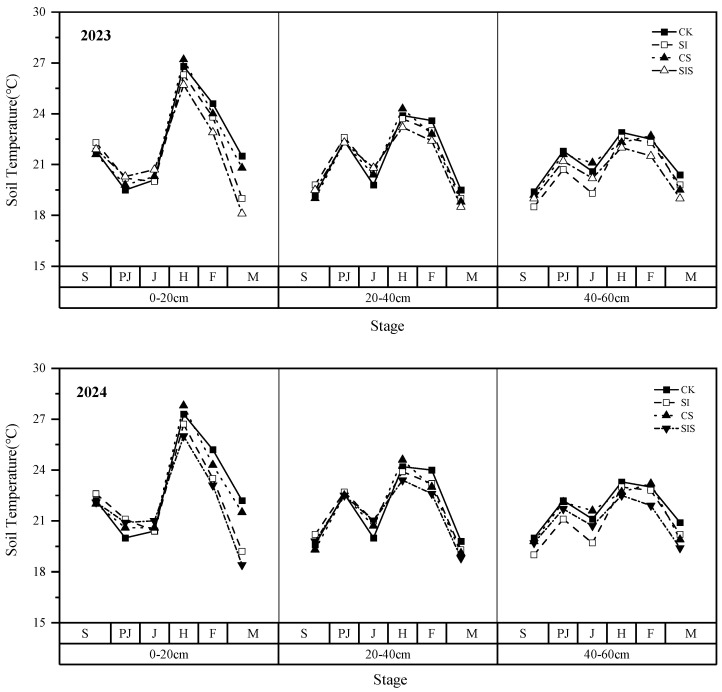
Soil temperature dynamics at different depths under each treatment.

Figure 7 presents soil temperature dynamics in 2023 and 2024. During the jointing stage and earlier, SI slightly increased soil temperature by 0–4.04% (2023) and 0–5.50% (2024) at 0–40 cm, whereas it decreased soil temperature in the 40–60 cm layer by 1.04–6.31% and 0.50–6.64%, respectively. During the heading stage, CS led to modest increases in soil temperature of 1.49–1.67% (2023) and 1.65–1.83% (2024) at 0–40 cm but reduced temperature in the 40–60 cm layer by 2.62% and 2.58%. SIS produced marginally lower soil temperatures across all layers than SI, with decreases of 2.11–2.65% in 2023 and 2.09–2.62% in 2024. At grain filling, Se treatments resulted in small reductions in soil temperatures of 2.61–4.74% (2023) and 1.70–4.78% (2024) relative to non-Se treatments. While supplemental irrigation increased soil temperature during the early growth stages, Se application appeared to moderate soil temperature later in millet stages. Overall, these practices contributed to more stable soil thermal conditions for millet growth.

(3) Soil Enzyme Activities

Urease, sucrase, and catalase are important factors influencing Rs. Urease supplies ammonium nitrogen, which promotes microbial growth and increases respiration. Sucrase provides microorganisms with carbon sources and energy, supporting microbial proliferation and driving aerobic Rs. Catalase decomposes hydrogen peroxide into water and oxygen, creating a safer microenvironment for microorganisms and helping maintain stable Rs. Appropriate supplemental irrigation regulates soil moisture, improves soil aggregate structure, and enhances microbial diversity and enzyme activity.

**Figure 8:**
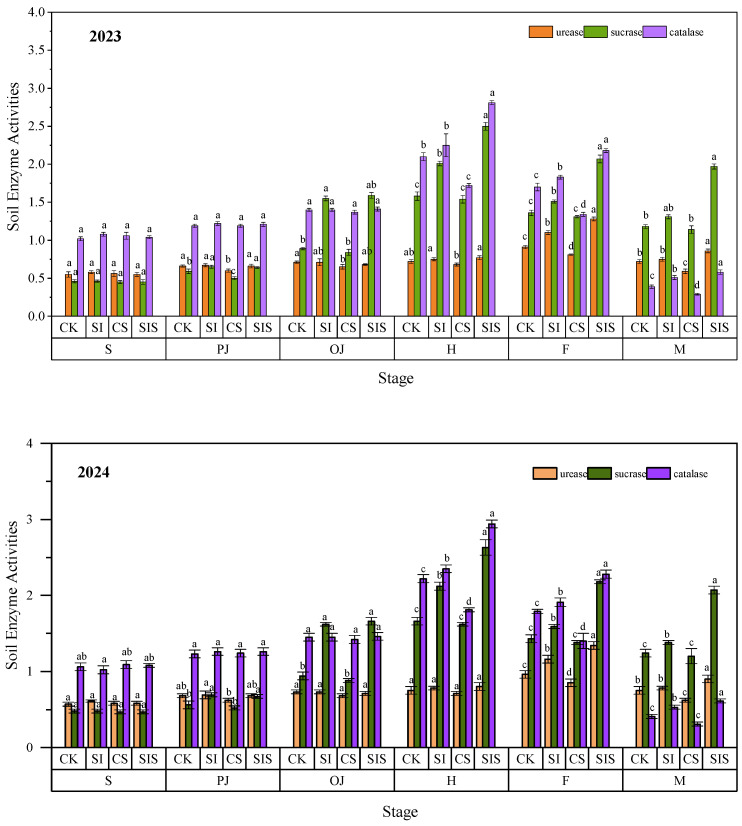
Dynamics of soil enzyme activities under different treatments. Data are presented as mean ± standard deviation (SD) from three replicates per treatment. Different letters in the columns indicate significant differences among supplemental irrigation and Se application treatments at *p* < 0.05 according to the LSD test. The units of soil urease, sucrase, and catalase are mg/(g·d), mg/(g·d), and ml/(g·h), respectively.

Soil enzyme activities under the different treatments showed dynamic changes during the millet growth period, characterized by an initial increase followed by a decline (Figure 8). Urease activity peaked at the filling stage, whereas sucrase and catalase activities were highest at heading. In both years, SI and CS had significant (*p* < 0.05) effects on urease activity at the filling and maturity stages. SI significantly increased (*p* < 0.05) soil sucrase activity from heading to maturity, and SIS notably enhanced sucrase activity throughout the entire growth period. Supplemental irrigation and Se application also had significant effects (*p* < 0.05) on catalase activity from heading to filling. Relative to CK, SI increased urease, sucrase, and catalase activities by 4.16–20.88%, 11.01–27.22%, and 7.14–30.77% in 2023 and by 4.00–20.83%, 11.19–27.71%, and 5.86–29.27% in 2024, respectively. In contrast, CS reduced the activities of the three enzymes by 5.55–18.06%, 2.53–3.68%, and 18.09–25.64% in 2023, and by 5.33–17.33%, 2.40–3.50%, and 18.46–24.39% in 2024, respectively. The combined SIS treatment substantially increased enzyme activities, with increases of 6.94–40.66%, 52.20–66.95%, and 28.23–48.72% in 2023 and 12.67–57.65%, 57.97–72.50%, and 62.43–96.77% in 2024. Supplemental irrigation enhanced soil enzyme activities, whereas Se application exerted suppressing effects. Overall, irrigation had a stronger influence on enzyme activities than Se fertilization. However, their combination generated a cooperative enhancement effect.

(4) Soil Organic Matter

**Figure 9:**
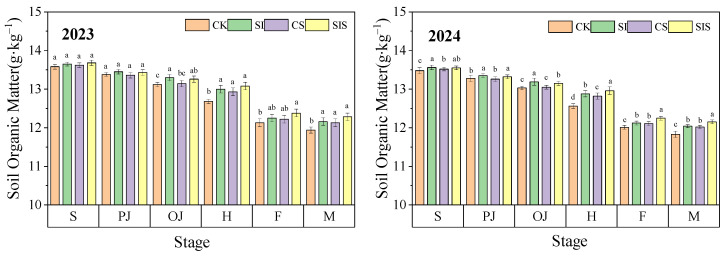
Dynamics of SOM under different treatments. Data are presented as mean ± standard deviation (SD) from three replicates per treatment. Different letters in the columns indicate significant differences among supplemental irrigation and Se application treatments at *p* < 0.05 according to the LSD test.

Soil organic matter (SOM) in all treatments gradually declined during the millet growth period, with the rate of decline first increasing and then slowing. As shown in Figure 9, the initial decline at the seedling stage was modest, followed by accelerated SOM loss from jointing to heading, corresponding to vigorous crop growth and high nutrient demand. During the filling stage, SOM decreased more slowly due to litter return from aboveground biomass and reduced decomposition associated with plant senescence. Supplemental irrigation and Se application influenced SOM content to varying degrees. Compared with CK, SOM content increased under SI, CS, and SIS by 0.51–2.52%, 0.61–1.06%, and 0.37–3.15% in 2023 and by 0.52–2.55%, 0.83–2.07%, and 0.30–3.18% in 2024, respectively. The SIS treatment showed a clear trend of further improving SOM content across both years.

### 2.6. Analysis of the Effects of Various Influencing Factors on Soil CO_2_Respiration Rate and Its Components in Millet Fields

**Figure 10:**
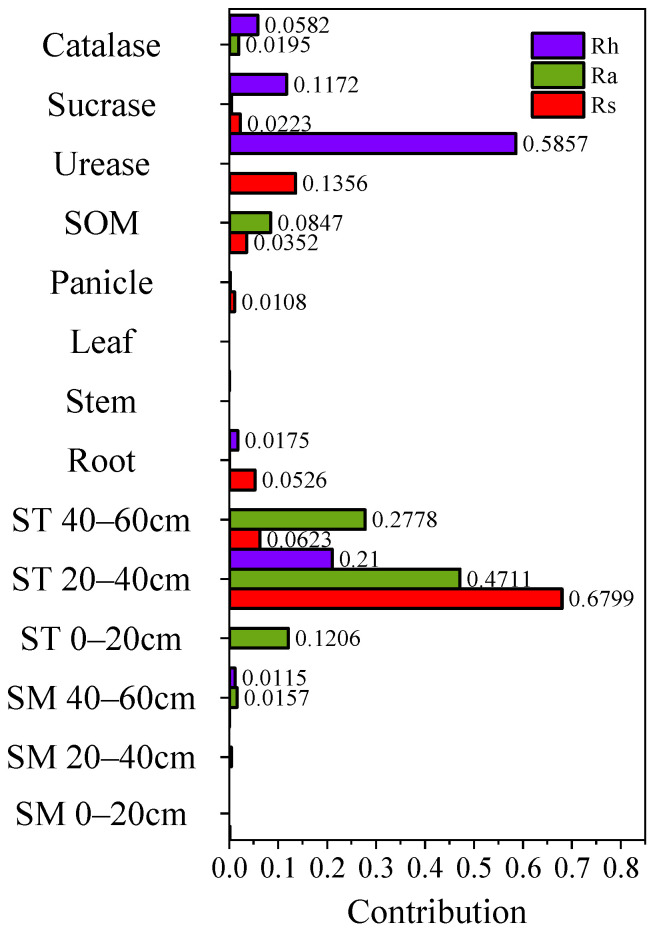
Contribution of influencing factors to Rs and its components in millet fields. These variables of ST 0–20 cm, ST 20–40 cm, ST 40–60 cm, SM 0–20 cm, SM 20–40 cm, and SM 40–60 cm refer to soil temperature at the soil depth of 0–20 cm, soil temperature at the soil depth of 20–40 cm, soil temperature at the soil depth of 40–60 cm, soil moisture at the soil depth of 0–20 cm, soil moisture at the soil depth of 20–40 cm, and soil moisture at the soil depth of 40–60 cm, respectively.

Figure 10 illustrates the feature weights of factors affecting Rs and its components in millet fields. Soil temperature at 20–40 cm, soil urease activity, and soil temperature at 40–60 cm together accounted for 87.78% of the variation in Rs, contributing 67.99%, 13.56%, and 6.23%, respectively. For Ra, soil temperature at 0–20, 20–40, and 40–60 cm explained 86.95% of its variation, with contributions of 47.11%, 27.78%, and 12.06%, respectively. For Rh, soil urease activity, soil temperature at 20–40 cm, and soil sucrase activity were the primary factors, contributing 58.57%, 21.00%, and 11.72%, respectively. Root biomass, SOM, soil sucrase activity, and panicle weight contributed 5.26%, 3.52%, 2.23%, and 1.08% to Rs, respectively. For Ra, SOM, soil catalase activity, and soil water content at 40–60 cm contributed 8.47%, 1.95%, and 1.57%, respectively. For Rh, soil catalase activity, root biomass, and soil water content at 40–60 cm accounted for 5.82%, 1.75%, and 1.15%, respectively. These results suggest that Ra is primarily associated with soil temperature, whereas Rh is mainly related to soil enzyme activity.

## 3. Discussion

### 3.1. Effects of Supplemental Irrigation and Se Application on the Dynamics of Rs Rate

Rs and its components under all treatments increased initially and then declined throughout the millet growth period, with Ra contributing the largest share. SI increased Rs at the jointing stage relative to CK, but Rh was lower under SI at maturity. This shift reflects the dynamic balance between soil moisture–temperature conditions and carbon supply [32].

Post-jointing supplemental irrigation improved soil hydrothermal conditions in the 0–40 cm layer, which likely promoted root growth and stimulated soil enzyme activities. Enhanced soil aeration under these conditions may have facilitated enzymatic transformations of organic substrates, contributing to higher urease and sucrase activities [33,34,35]. These biochemical changes could accelerate SOM decomposition, fostering a synergistic “water–thermal–aeration” effect that elevated Rs during the late jointing stage [36,37]. Conversely, Se application at the heading stage reduced soil temperature in the 40–60 cm layer during the filling stage. Lower soil temperatures in sub-soil depth may slow microbial metabolism and diminish enzyme-mediated breakdown of organic materials, thereby restricting substrate availability for Rh [38]. Simultaneously, root senescence during the filling stage naturally weakens Ra [39]. The combined decline in Ra and Rh likely contributed to the observed reduction in Rs at the maturity stage. At the maturity stage, Rh under CK and SI exceeded Se treatments. Soil moisture at 0–20, 20–40, and 40–60 cm under CK and SI was lower by 5.99–6.44%, 8.69–11.55%, and 6.99–9.12% in 2023, and by 5.63–6.01%, 8.29–11.12%, and 2.28–6.27% in 2024, respectively. Under these drier conditions, fungal-mediated lignin decomposition may enhance Rh [40].

The observed diurnal patterns of Rs and its components varied across crop growth stages. Similar dynamical variability has been attributed to management-induced changes in plant physiology, as well as microbial activity, which was reported under contrasting irrigation and nitrogen regimes in Indian agroecosystems [41]. Further studies across diverse climates have demonstrated that diurnal Rs fluctuations are jointly regulated by soil moisture, temperature, and nutrient availability [42,43]. Therefore, the accurate modeling of Rs requires an integrated consideration of phenological development and environmental constraints.

### 3.2. Synergistic Effect of Supplemental Irrigation and Se Application on the Rs Environment in Millet Fields

Under the combined influence of residual soil moisture from post-jointing irrigation and the photosynthetic enhancement induced by Se application, the highest Rs under SIS occurred at heading. SI maintained higher soil moisture and enzyme activity at heading, elevating Rh. CS increased Ra by enhancing photosynthesis.

SI increased soil moisture and enzyme activity [44], promoting plant growth and mineralization of SOM [45]. Therefore, cumulative CO_2_ emissions under SI increased (*p* < 0.05) by 18.12% in 2023 and 18.40% in 2024 relative to CK. CS enhanced photosynthetic efficiency and grain formation: CS increased panicle dry matter by 26.04% (*p* < 0.5). However, Se slightly suppressed soil enzyme activity, slowing organic matter decomposition and lowering root-derived carbon loss. By inhibiting Ra, CS reduced cumulative CO_2_ emissions by 10.02–10.43% (*p* < 0.05). The SIS treatment resulted in moderate emission increases (16.84% and 16.77%) while retaining yield benefits. The SIS treatment thus reflects a synergistic pattern of “water conservation–fertility enhancement–yield improvement”. Nevertheless, due to the limited spatial scale and single-site experiment, these findings should be regarded as preliminary indications of potential mitigation effects. Broader application in agricultural systems requires multi-site, long-term validation across diverse experiment conditions.

The influence of soil moisture on enzyme activity is also mediated by temperature. As humidity increases, the dominant control shifts from moisture to temperature [46]. Se application further affects grain development and enzyme activity in complex ways. Se may reduce reactive oxygen species damage by modulating catalase activity, thus supporting assimilate translocation to grains [47], yet excessive Se may suppress microbial diversity [48]. Therefore, optimizing thresholds for supplemental irrigation and Se inputs requires careful management to balance soil carbon dynamics, yield gains, and emission mitigation.

The relatively small magnitude of SOM variation can be attributed to several factors. The SOM accumulation rate is generally slow. The duration of the current experiment was insufficient to detect large measurable changes [49]. SOM was influenced by complex interactions between organic inputs, microbial decomposition, and soil physicochemical properties [50]. Additionally, temperature, moisture, and native SOM levels in the study region may have constrained the rate of SOM formation and stabilization [51]. Therefore, the small differences observed in SOM mainly result from its natural stability and slow response to management practices. Although the percentage differences in SOM among treatments were relatively small, their agronomic relevance should not be ignored. The slight increases in SOM can still improve soil structure, enhance water and nutrient retention, and stimulate microbial activity, thereby creating a more favorable environment for root development and plant growth [52]. Especially in long-term agricultural processes, the slow accumulation of SOM caused by integrated management (over successive cropping cycles) is helpful in improving soil fertility and resilience against soil degradation [53]. In this study, the observed trends suggest that supplemental irrigation combined with selenium application promotes a gradual accumulation of SOM, which has positive significance for achieving sustainable agricultural development.

Validation through experiments with a larger number of plots and replications is also necessary to confirm the feasibility and promote the production model of millet farmland management strategies.

### 3.3. Balancing Yield Enhancement and Carbon Emission Mitigation in Millet Production

Supplemental irrigation at the jointing stage improved soil moisture during drought-prone periods, creating favorable conditions for moisture conservation, aeration, and decomposition, which collectively supported yield formation [54,55]. Se application facilitated the translocation of assimilates to grains by reducing reactive oxygen species damage and suppressing soil enzyme activity [56,57]. By optimizing carbon allocation across the root–soil–microbe–grain system, the CS treatment increased yield by 28.53% in 2023 and 28.02% in 2024, while reducing carbon emissions by 1.08% and 1.38% relative to CK. CS thus alleviated drought stress and improved yield formation while contributing to emission mitigation.

## 4. Materials and Methods

### 4.1. Study Area

**Figure 11:**
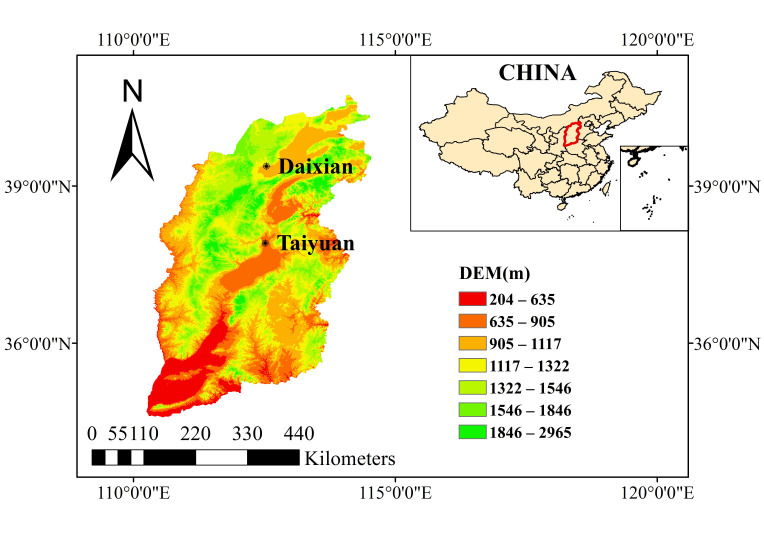
The geographical location of the study area.

**Figure 12:**
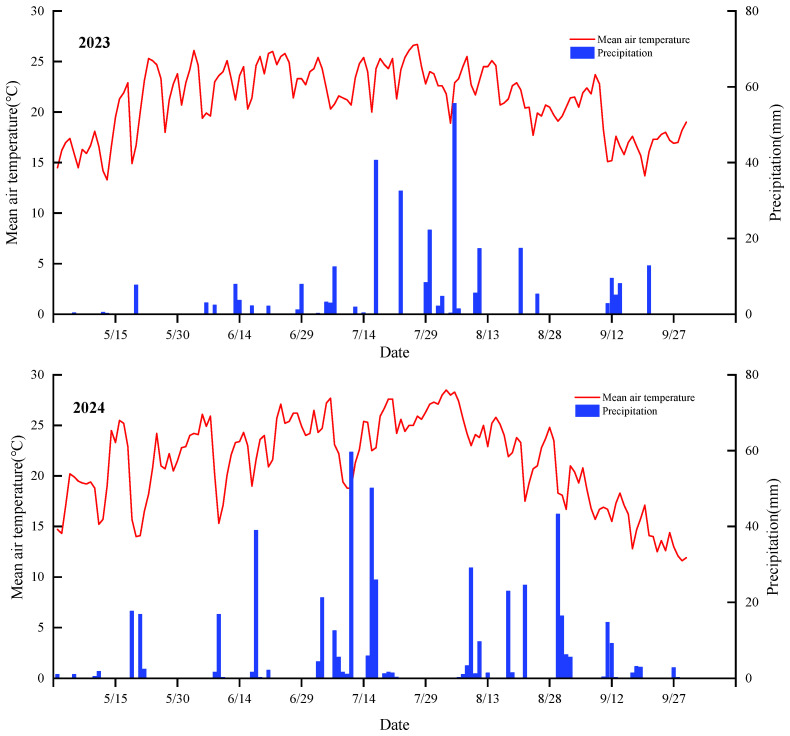
Mean air temperature and precipitation during the millet growth periods in 2023 and 2024.

The field experiment was conducted in the Daixian District (39°4′12″ N, 112°56′47″ E), Xinzhou, Shanxi Province, during 2023 and 2024 (Figure 11). The region has a semi-arid continental monsoon climate, with a mean annual precipitation of approximately 424 mm and annual evapotranspiration (ET) of 1760 mm. The mean annual temperature is 8.5 °C, with an extreme maximum of 38.9 °C and an extreme minimum of −24.5 °C. The frost-free period is about 160 days, and the effective accumulated temperature (5–25 °C) is sufficient for millet production. Precipitation from July to September accounts for roughly 70% of the annual total. The meteorological variables during the millet growth periods in 2023 and 2024 are shown in Figure 12. Basic soil properties are summarized in Table 2.

### 4.2. Experimental Design

The millet variety Jingu No. 53 was used for field experiments from May 6 to September 8 in 2023 and from May 13 to September 12 in 2024. Light rainfall (~5 mm) during the sowing period in both years supported adequate germination. Four treatments were established to evaluate the effects of supplemental irrigation and Se application, namely, conventional rainfed (CK), supplemental irrigation (SI), rainfed + Se addition (CS), and supplemental irrigation + Se addition (SIS), each with three replicates. The SIS treatment considered the synergistic effect between supplemental irrigation and Se addition. Based on previous findings on optimal supplemental irrigation and foliar Se application for millet yield [29,58], 50 mm of irrigation was applied at the post-jointing stage, and foliar Na_2_SeO_3_ (67.84 g·hm^−2^) was applied at the heading stage. Other field management practices, including fertilization, pesticide application, and weed control, followed local agricultural standards.

**Figure 13:**
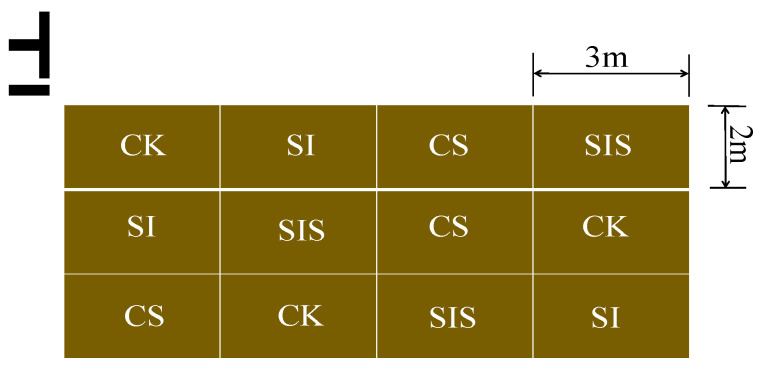
Field experimental layout of millet.

Each plot measured 6 m^2^ (2 m × 3 m), with rows spaced 30 cm apart and plants spaced 10 cm apart. As shown in Figure 13, the field layout followed an orthogonal design to systematically evaluate the main effects of the key factors. To ensure the reliability of the experiment and minimize the impact of micro-environmental variations, the specific positions of the treatment plots were randomized within the constraints of the orthogonal array. To prevent lateral water movement, plastic impermeable barriers were installed vertically to a depth of 60 cm between adjacent plots. Basal fertilization was applied before sowing using a compound fertilizer (N:P_2_O_5_:K_2_O = 18:18:18) at 600 kg·hm^−2^. Growth stages for millet across both years are summarized in Table 3.

### 4.3. Measurement Methods

#### 4.3.1. Rs Rate and CO_2_ Emission Flux

Rs was measured using a custom-built transparent static chamber made of See (PVC) connected to a portable infrared CO_2_ analyzer (Model FS-3080D, Shijiazhuang Fansheng Technology Limited Company, Shijiazhuang, China). The system consisted of a base collar and a removable transparent cylindrical chamber. The base collar (20 cm diameter) was inserted 10 cm into the soil to ensure an airtight seal. Both components were fitted with a grooved, water-filled channel to create a gas-tight interface. The removable chamber was connected to the analyzer for gas sampling.

**Figure 14:**
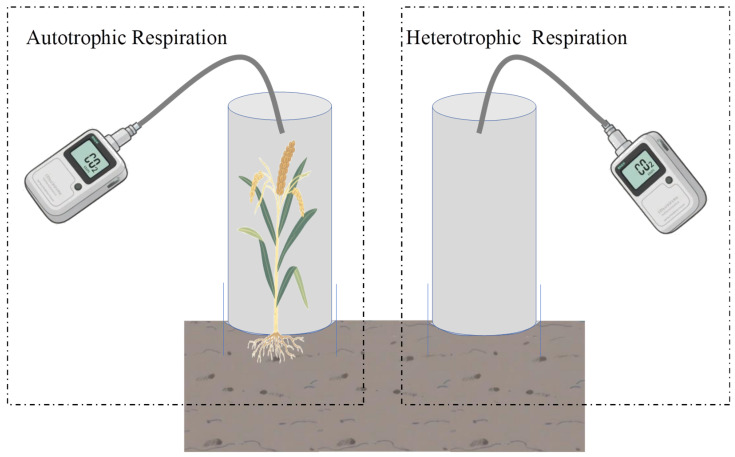
The measurement system for Rs in the millet field.

Ra was measured by placing the chamber over an intact plant, whereas Rh was measured by placing the chamber directly on bare soil. The chamber height was adjusted according to the average millet plant height at each growth stage, which can fully cover the canopy. The applying chamber heights were 40 cm, 80 cm, 120 cm, 160 cm, and 180 cm at the seedling, jointing, heading, filling, maturity stages, respectively. The measurement system is shown in Figure 14.

Measurements were taken from 6:00 to 20:00 at 2-h intervals on one representative plant per treatment, with three replicates. Rs was monitored every 15–20 days throughout the growing season. Previous studies reported that soil moisture typically reached a steady state within one to several days after major rainfall or irrigation events [59,60]. To minimize the influence of abrupt changes in soil moisture on short-term measurement outcomes, all sampling days were scheduled to occur at least 48 h after any substantial rainfall or irrigation event. Before each measurement, the system was preheated for 15 min to ensure uniform gas mixing. The analyzer automatically recorded CO_2_ concentration at 2 min intervals, and three consecutive readings were taken per measurement.

Rs rate (S, mg·m^−2^·h^−1^) and cumulative CO_2_ emissions (F, g·m^−2^) were calculated using Formulas (1) and (2):

(1) $$S=\frac{273\rho HPd_{c}}{\left(273+T\right)P_{0}d_{t}}$$

where ρ is the density of CO_2_ under standard conditions (1.963 g·L^−1^); H is the effective height of the chamber (m); P_0_ is the atmospheric pressure under standard conditions (1.01 × 10^5^ Pa); P and T are the actual air pressure (Pa) and temperature (°C) inside the chamber during measurement, respectively; and dc/dt is the change rate of CO_2_ concentration over time inside the static chamber.

(2) $$F=\frac{86400}{10^{6}}\times \sum_{i=1}^{n}\frac{\left(S_{i+1}+S_{i}\right)}{2}\times \left(t_{i+1}-t_{i}\right)\times 44$$

where (t_i + 1_ − t_i_) is the time interval between consecutive measurements (days), n is the measurement count, and 44 is the molar mass of CO_2_.

#### 4.3.2. Soil Temperature and Soil Moisture Content

Soil temperature (ST, °C) and soil moisture (SM, m^3^·m^−3^) were recorded concurrently with respiration measurements. Using the three-point sampling method, soil temperature and moisture at depths of 0–20, 20–40, and 40–60 cm were measured at 8:00 using a pre-installed soil temperature and moisture monitoring system (Delta-T Wet, Shaanxi Hemurun Agricultural Services Limited Company, Xi’an, China).

#### 4.3.3. Soil Enzyme Activity and SOM

Soil samples for enzyme activity and SOM determination were collected using an S-shaped sampling pattern at 0–20, 20–40, and 40–60 cm during the seedling, jointing, heading, filling, and maturity stages. Each composite sample was divided: one portion was stored at 4 °C for enzyme activity analysis, and the other was air-dried and sieved (0.15 mm) for SOM analysis. Urease activity was measured using the sodium phenol–sodium hypochlorite colorimetric method [61] and expressed as mg NH_3_–N g^−1^·d^−1^. Sucrase activity was determined using the 3,5-dinitrosalicylic acid method [62]. Catalase activity was quantified by potassium permanganate titration and expressed as mL 0.1 mol·L^−1^ KMnO_4_ g^−1^·h^−1^ [63]. SOM was measured by potassium dichromate oxidation with external heating [64].

#### 4.3.4. Dry Matter Weight

At each growth stage, three representative plants per treatment were sampled for dry matter determination. Roots were collected from 0 to 20, 20 to 40, and 40 to 60 cm using a 10 cm diameter root auger (KHT-016, Jintan Kanghua Electronic Instrument Factory, Jintan, China). Excavated roots were washed in 0.5 mm nylon mesh bags [65]. The roots, stems, leaves, leaf sheaths, and panicles were oven-dried after an initial 30 min treatment at 105 °C to deactivate enzymes, followed by drying at 80 °C to constant weight. Dry matter was measured to 0.001 g precision, and total plant dry matter was calculated as the sum of all components.

#### 4.3.5. Yield

At physiological maturity, all grains in each treatment were harvested, dried, and threshed to determine yield (Y, kg·ha^−1^).

#### 4.3.6. WUE

Water consumption (ET, mm) was calculated using the water balance method [6], considering changes in soil moisture (W), precipitation (P), and irrigation (I). Field drainage, runoff, deep percolation, and groundwater recharge were assumed negligible.

ET = P + I − 10 × D × H × (Wt + 1 − Wt)(3)

where D is the soil bulk density (1.3 g·cm^−3^); H (cm) is the millet planning–wetting layer depth ranging from 0 to 60 cm; Wt is the moisture content (%) of each soil layer from 0 to 60 cm in the end of period; and W_t + 1_ is the moisture content (%) of each soil layer from 0 to 60 cm in the beginning of the period.

The WUE (kg·ha^−1^ ·mm^−1^) is the Y per unit ET and calculated using Formula (4).

WUE = Y/ET(4)

### 4.4. Data Analysis and Processing

#### 4.4.1. Feature Weights Assessment

In this study, the Classification and Regression Tree (CART) algorithm was used to quantify the feature weights of several independent variables, including soil moisture, soil temperature, dry biomass of different plant organs, soil organic matter (SOM), and soil enzyme activities. The CART algorithm was trained using the complete dataset, with the target variable measured in the field. The model parameters, including tree depth and minimum samples per leaf, were optimized using k-fold cross-validation (k = 5) to prevent overfitting and ensure generalizability. The CART algorithm is a non-parametric supervised learning method that recursively partitions the data into homogeneous subsets based on the variance reduction criterion for regression tasks [66]. The feature weight indicates the relative importance for interpreting internal mechanisms in complex models and identifying variables that contribute to target index [67]. The feature weight of each variable in this study expresses its influence on total Rs and on its autotrophic and heterotrophic components in the millet agroecosystem. A higher feature weight indicates a stronger effect on Rs.

#### 4.4.2. Statistical Analysis

All measurements were recorded and preliminarily analyzed using Excel 2016 (Microsoft, Redmond, Washington, WA, USA). To visualize the effects of supplemental irrigation and Se application on Rs rate, CO_2_ emissions, dry matter, soil enzyme activity, SOM, ET, yield, and WUE in the same period, the statistical model of SPSS 24.0 (IBM SPSS Statistics, Armonk, New York, NY, USA) was used for the comparison between treatments. One-way ANOVA in SPSS 24.0 was chosen to determine the significant differences among supplemental irrigation and Se application treatments, and post hoc comparisons (Fisher’s LSD) were adopted for multiple comparisons test (*p* < 0.05). Significance levels of *p* < 0.05 indicated significant difference. For each soil layer, the average SM and ST in the same period from the three replicates at different soil depths among the four treatments were calculated using Microsoft Office 2016. These comparisons of SM and ST profiles were simply calculated by range amplitude.

Figures were generated using Origin 2024 (Origin Lab Corporation, Northampton, MA, USA).

## 5. Conclusions

This study examined the effects of supplemental irrigation and Se application in millet fields on Rs rate and its components, cumulative CO_2_ emissions, dry matter accumulation, ET, yield, WUE, soil moisture, soil temperature, SOM, and soil enzyme activity under the specific edaphoclimatic conditions of the experimental site. Ra was the predominant component of Rs in each treatment. Supplemental irrigation increased CO_2_ emissions, whereas Se application reduced them. The CS treatment produced the lowest cumulative CO_2_ emissions at 4233 and 4009 g·m^−2^ in 2023 and 2024. Decision Tree analysis indicated that Ra was mainly influenced by soil temperature (feature weight = 86.95%), whereas Rh was primarily regulated by soil enzyme activity (feature weight = 76.11%). Overall, CS played a significant role in emission mitigation and maintained the same yield with rain-fed millet. The SIS treatment achieved the highest yield and maintained a lower Rs than SI. These findings suggest that an appropriate combination of supplemental irrigation and Se fertilization could support millet production while maintaining an environmentally friendly farmland system. The study was limited by its spatial scale and single-site design. Further studies with a larger number of plots across diverse sites are necessary to confirm the broader feasibility of millet farmland management strategies.

## Figures and Tables

**Table 1:** Water consumption, yield, and WUE of millet under different treatments.

Year	Items	Treatments			
		CK	SI	CS	SIS
2023	ET (mm)	248.3 ± 2.25 c	268.50 ± 1.50 b	285.7 ± 2.50 a	287.9 ± 1.05 a
	Yield (kg·ha^−1^)	3920.5 ± 25.24 d	4526.9 ± 25.02 c	5038.0 ± 50.41 b	5624.3 ± 16.22 a
	WUE (kg·ha^−1^·mm^−1^)	15.79 ± 0.05 d	16.86 ± 0.01 c	17.64 ± 0.04 b	19.53± 0.04 a
2024	ET (mm)	256 ± 6.44 c	275.2 ± 4.22 b	291.4 ± 1.67 a	293.2 ± 2.95 a
	Yield (kg·ha^−1^)	4059.2 ± 107.37 d	4702.5 ± 81.46 c	5196.7 ± 51.96 b	5820.0 ± 58.20 a
	WUE (kg·ha^−1^·mm^−1^)	15.86 ± 0.09 d	17.09 ± 0.10 c	17.83 ± 0.20 b	19.85 ± 0.00 a

Note: Data are presented as mean ± standard deviation (SD) based on three replicates per treatment. Different letters within the same row indicate significant differences among SI and CS treatments according to the LSD (*p* < 0.05).

**Table 2:** Soil physical–chemical properties in the study area.

Items	2023	2024
Field capacity (%)	26.5% ± 1.6	26.5 ± 1.8
Total N content (g·kg^−1^)	0.69 ± 0.03	0.83 ± 0.04
Soil organic matter content (g·kg^−1^)	13.9 ± 0.1	13.68 ± 0.1
Available phosphorus content (mg·kg−1)	15.8 ± 0.1	13.29 ± 0.2
Available potassium content (mg·kg^−1^)	146.9 ± 6.5	159.0 ± 7.4
pH	6.5 ± 0.06	6.6 ± 0.05
Soil texture	Loam	Loam

**Table 3:** Growth stages of millet in the field experiment.

Stage	2023	2024
Seedling	5.6–6.14	5.13–6.14
Jointing	6.15–7.18	6.15–7.18
Heading	7.19–8.4	7.19–8.4
Filling	8.5–8.21	8.5–8.21
Maturity	8.22–9.8	8.22–9.12

## Data Availability

The original contributions presented in this study are included in the article. Further inquiries can be directed to the corresponding author.

## References

[1] Liu J.L., Chang L., Duan X.L., Wang W.J., Sun H. (2022). Foxtail Millet: Production Status, Advances in Health Benefits and Its Mechanism. Sci. Technol. Food Ind..

[2] Suri S., Balasaheb K.S., Yadav D.K., Malakar S., Choudhary P., Mohapatra A., Dhurve P. (2024). Overview of Millet Proteins: Quality Characteristics, Effect of Thermal/Non-Thermal Processing and Applications. Food Biosci..

[3] Zhang P.P., Wang B.L., Guo Y.N., Wang T., Qian W., Yan L., Hao L., Wu H.P., Wang X.L., Xiong Z. (2024). Identification of Drought-Resistant Response in Proso Millet (*Panicum miliaceum* L.) Root through Physiological and Transcriptomic Analysis. Plants.

[4] Ibrahim A., Abaidoo R.C., Fatondji D., Opoku A. (2015). Hill Placement of Manure and Fertilizer Micro-Dosing Improves Yield and Water Use Efficiency in the Sahelian Low Input Millet-Based Cropping System. Field Crops Res..

[5] Zhou S.W., Wu Y.Z., Wang C., Lu H.Y., Zhang Z.C., Liu Z.J., Lei Y.D., Chen F. (2024). Projection of Future Drought Impacts on Millet Yield in Northern Shanxi of China Using Ensemble Machine Learning Approach. Comput. Electron. Agric..

[6] Nie T.Z., Li J.F., Jiang L.L., Zhang Z.X., Chen P., Li T.C., Dai C.L., Sun Z.Y., Yin S., Wang M.X. (2024). Optimizing Irrigation and Nitrogen Application to Enhance Millet Yield, Improve Water and Nitrogen Use Efficiency and Reduce Inorganic Nitrogen Accumulation in Northeast China. Plants.

[7] Luo P.Y., Han X.R., Wang Y., Han M., Shi H., Liu N., Bai H.Z. (2015). Influence of Long-Term Fertilization on Soil Microbial Biomass, Dehydrogenase Activity, and Bacterial and Fungal Community Structure in a Brown Soil of Northeast China. Ann. Microbiol..

[8] Tang J., Wang J.J., Li Z.Y., Wang S.N., Qu Y.K. (2018). Effects of Irrigation Regime and Nitrogen Fertilizer Management on CH_4_, N_2_O and CO_2_ Emissions from Saline–Alkaline Paddy Fields in Northeast China. Sustainability.

[9] Xu Y.N., Sheng J., Zhang L.P., Sun G.F., Zheng J.C. (2025). Organic Fertilizer Substitution Increased Soil Organic Carbon through the Association of Microbial Necromass C with Iron Oxides. Soil Tillage Res..

[10] Bond-Lamberty B., Thomson A. (2010). Temperature-Associated Increases in the Global Soil Respiration Record. Nature.

[11] Jiang J.S., Guo S.L., Zhang Y.J., Liu Q.F., Wang R., Wang Z.Q., Li N.N., Li R.J. (2015). Changes in Temperature Sensitivity of Soil Respiration in the Phases of a Three-Year Crop Rotation System. Soil Tillage Res..

[12] Gao X.L., Zhao N., Lu Y.H., Han X., Yang Z.P. (2022). Effects of Supplementary Irrigation on Soil Respiration of Millet Farmland in a Semi-Arid Region in China. Atmosphere.

[13] Yang J.Y., Zhang F.B., Li Y.Y., Gao J.X., Deng L., Shi W.Y., Shen N., Yang M.Y. (2025). Moisture Conditions Trigger Different Response Patterns of Soil Respiration to Biochar-Induced Changes in Soil Vertical Water Content and Temperature Based on a Three-Year Field Observation Study. Agric. Ecosyst. Environ..

[14] He L.P., Lin J.J., Lan B., Duan L.Y., Xu Z.J., Liao Y.H. (2021). The Effect of Increased Nitrogen Levels on Soil CO_2_ Emission Caused by Microbial Respiration in the Riparian Zone of the Three Gorges Reservoir. Appl. Ecol. Environ. Res..

[15] Li Y.C., Hou C.C., Wang Q.B., Chen Y.Y., Ma J.M., Mohammad Z. (2016). Effect of No-Till Farming and Straw Mulchon Spatial Variability of Soil Respirationin Sloping Cropland. Pol. J. Environ. Stud..

[16] Li C.X., Wang G.S., Han Q.S., Sun J.S., Ning H.F., Feng D. (2023). Soil Moisture and Water-Nitrogen Synergy Dominate the Change of Soil Carbon Stock in Farmland. Agric. Water Manag..

[17] Nissan A., Alcolombri U., Peleg N., Galili N., Jimenez-Martinez J., Molnar P., Holzner M. (2023). Global Warming Accelerates Soil Heterotrophic Respiration. Nat. Commun..

[18] Ren C.J., Wang T., Xu Y.D., Deng J., Zhao F.Z., Yang G.H., Han X.H., Feng Y.Z., Ren G.X. (2018). Differential Soil Microbial Community Responses to the Linkage of Soil Organic Carbon Fractions with Respiration across Land-Use Changes. For. Ecol. Manag..

[19] Shen H.Z., Li S.L., Sun K.X., Gao Y.H., Liu Y.X., Ma X.Y. (2023). Integrated Impacts of Irrigation and Nitrogen Management for Balancing Winter Wheat Yield and Greenhouse Gas Emissions. Crop Environ..

[20] Liu X.J., Zhang Y., Han W.X., Tang A.H., Shen J.L., Cui Z.L., Vitousek P., Erisman J.W., Goulding K., Christie P. (2013). Enhanced Nitrogen Deposition over China. Nature.

[21] Wang M., Liu X.T., Zhang J.T., Li X.J., Wang G.D., Li X.Y., Lu X.R. (2014). Diurnal and Seasonal Dynamics of Soil Respiration at Temperate *Leymus chinensis* Meadow Steppes in Western Songnen Plain, China. Chin. Geogr. Sci..

[22] Wang W., Guo L.P., Li Y.C., Su M., Lin Y.B., De Perthuis C., Ju X.T., Lin E., Moran D. (2015). Greenhouse Gas Intensity of Three Main Crops and Implications for Low-Carbon Agriculture in China. Clim. Change.

[23] Yan W.M., Zhong Y.Q.W., Liu W.Z., Shangguan Z. (2021). Asymmetric Response of Ecosystem Carbon Components and Soil Water Consumption to Nitrogen Fertilization in Farmland. Agric. Ecosyst. Environ..

[24] Zhang P.A., Li L., Fu Q., Du C.Z., Yang A.Z., Sun N., Wang L.H., Li M. (2025). Balancing Soil Carbon Emissions and Productivity in Maize Agroecosystems through Nitrogen, Biochar, and Straw Regulation. Ind. Crops Prod..

[25] Yang S.H., Xiao Y.N., Xu J.Z. (2018). Organic Fertilizer Application Increases the Soil Respiration and Net Ecosystem Carbon Dioxide Absorption of Paddy Fields under Water-Saving Irrigation. Environ. Sci. Pollut. Res. Int..

[26] Li X.J., Sun J.J., Li W.S., Gong Z.Q., Jia C.Y., Li P.J. (2022). Effect of Foliar Application of the Selenium-Rich Nutrient Solution on the Selenium Accumulation in Grains of Foxtail Millet (Zhangzagu 10). Environ. Sci. Pollut. Res. Int..

[27] Zhou W.X., Duan Y.Y., Zhang Y.J., Wang H., Huang D.H., Zhang M.D. (2021). Effects of Foliar Selenium Application on Growth and Rhizospheric Soil Micro-Ecological Environment of *Atractylodes macrocephala* Koidz. S. Afr. J. Bot..

[28] Zhang L., Wang Q., Lv J.P., Zhang C. (2023). The Response of Soil Respiration to Land-use Change Depends on Soil Microbial Community Being Regulated by Edaphic Factors in the Loess Plateau, China. Land Degrad. Dev..

[29] Gao X.L., Ma J.J., Jia Y.R., Liu E.K., Song L.L. (2021). The Effects of Deficit Irrigation Scheduling on Water Consumption and Water Use Efficiency of Millet in the Northern Shanxi Province. J. Irrig. Drain..

[30] Keskinen R., Räty M., Yli-Halla M. (2011). Selenium Fractions in Selenate-Fertilized Field Soils of Finland. Nutr. Cycl. Agroecosyst..

[31] Deng X.F., Liu K.Z., Li M.F., Zhang W., Zhao X.H., Zhao Z.Q., Liu X.W. (2017). Difference of Selenium Uptake and Distribution in the Plant and Selenium Form in the Grains of Rice with Foliar Spray of Selenite or Selenate at Different Stages. Field Crops Res..

[32] Wang H.H., Huang W.D., He Y.Z., Zhu Y.Z. (2023). Effects of Warming and Precipitation Reduction on Soil Respiration in Horqin Sandy Grassland, Northern China. CATENA.

[33] Bünemann E.K., Bongiorno G., Bai Z., Creamer R.E., De Deyn G., de Goede R., Fleskens L., Geissen V., Kuyper T.W., Mäder P. (2018). Soil quality—A critical review. Soil Biol. Biochem..

[34] Abbas F., Hammad H.M., Ishaq W., Farooque A.A., Bakhat H.F., Zia Z., Fahad S., Farhad W., Cerdà A. (2020). A review of soil carbon dynamics resulting from agricultural management practices. Environ. Manag..

[35] Li X.D., Su L.B., Jing M., Wang K.Q., Song C.G., Song Y.L. (2025). Nitrogen addition restricts key soil ecological enzymes and nutrients by reducing microbial abundance and diversity. Sci. Rep..

[36] Dijkstra F.A., Carrillo Y., Pendall E., Morgan J.A. (2013). Rhizosphere priming: A nutrient perspective. Front. Microbiol..

[37] Kuzyakov Y., Blagodatskaya E. (2015). Microbial hotspots and hot moments in soil: Concept & review. Soil Biol. Biochem..

[38] Deng G.G., Fan Z.Q., Wang Z.Y., Peng M. (2025). Dynamic role of selenium in soil-plant-microbe systems: Mechanisms, biofortification, and environmental remediation. Plant Soil.

[39] Farooq M., Wahid A., Kobayashi N., Fujita D., Basra S.M.A. (2009). Plant drought stress: Effects, mechanisms and management. Agron. Sustain. Dev..

[40] Jia S.X., Zhou X.H., Fu Y.L., Zhou G.Y., Zhou L.Y., Wang X.X., Jiang Z., Sardans J., Penuelas J. (2025). Microbial Life History Mediates the Drought-Induced Decrease in Wood Decomposition in Subtropical Forests. Ecol. Lett..

[41] Sourav S.K., Subbarayappa C.T., Hanumanthappa D.C., Mudalagiriyappa, Vazhacharickal P.J., Mock A., Ingold M., Buerkert A. (2023). Soil Respiration under Different N Fertilization and Irrigation Regimes in Bengaluru, S-India. Nutr. Cycl. Agroecosyst..

[42] Lu N., Liu X.R., Du Z.L., Wang Y.D., Zhang Q.Z. (2014). Effect of Biochar on Soil Respiration in the Maize Growing Season after 5 Years of Consecutive Application. Soil Res..

[43] Singla A., Iwasa H., Inubushi K. (2014). Effect of Biogas Digested Slurry Based-Biochar and Digested Liquid on N_2_O, CO_2_ Flux and Crop Yield for Three Continuous Cropping Cycles of Komatsuna (*Brassica rapa* Var. Perviridis). Biol. Fertil. Soils.

[44] Chen L., Deng X.Y., Duan H.X., Tan X.M., Xie X.B., Pan X.H., Guo L., Gao H., Wei H.Y., Zhang H.C. (2023). Water Management Can Alleviate the Deterioration of Rice Quality Caused by High Canopy Humidity. Agric. Water Manag..

[45] Wang Y.Y., Wang W.D., Zheng M.Y., Ou X.Q., Zheng H.F. (2024). Effects of nitrogen application and irrigation treatment on soil organic carbon components and enzyme activities in wheat field. J. Environ. Eng. Technol..

[46] Kang N., Pacholski A.S. (2022). Soil Moisture and Temperature Effects on Granule Dissolution and Urease Activity of Urea with and without Inhibitors—An Incubation Study. Agriculture.

[47] Sobucki L., Ferraz Ramos R., Sobucki V., Pawlowski E., Kaiser D.R., Jacques R.J.S., Antoniolli Z.I., Pozzobon M.D., Schenato R.B., Daroit D.J. (2024). Sensitivity of Microbiological Properties of a Rhodic Ferralsol in Response to Management and Environmental Variables in Subtropical Brazil. Commun. Soil Sci. Plant Anal..

[48] Cao C.L., Lü H.Q., Hao Z.P., Gao X. (2021). The effects of exogenous selenium on photosynthetic characteristics, selenium accumulation in grain, yield and quality of “jin tartary buckwheat 5”. Soil Fertil. Sci..

[49] Six J., Bossuyt H., Degryze S., Denef K. (2004). A history of research on the link between (micro)aggregates, soil biota, and soil organic matter dynamics. Soil Tillage Res..

[50] Kemmitt S.J., Wright D., Goulding K.W.T., Jones D.L. (2006). pH regulation of carbon and nitrogen dynamics in two agricultural soils. Soil Biol. Biochem..

[51] Conant R.T., Cerri C.E., Osborne B.B., Paustian K. (2017). Grassland management impacts on soil carbon stocks: A new synthesis. Ecol. Appl..

[52] Lal R. (2004). Soil carbon sequestration impacts on global climate change and food security. Science.

[53] Hinojosa M.B., García-Ruíz R., Viñegla B., Carreira J.A. (2004). Microbiological rates and enzyme activities as indicators of functionality in soils affected by Aznalcóllar toxic spill. Soil Biol. Biochem..

[54] Araujo M.A., Melo A.A.R., Silva V.M., Reis A.R.D. (2023). Selenium enhances ROS scavenging systems and sugar metabolism increasing growth of sugarcane plants. Plant Physiol. Biochem..

[55] Soliman E.R.S., Abdelhameed R.E. (2025). Selenium Seed Priming Adjusts Photosynthesis, Metabolic Constituents and Gene Expression Profiling in *Vicia faba* L to Outstand Lead Stress. J. Soil Sci. Plant Nutr..

[56] Hu L., Zhang B.J., Wu D.S., Liu Y., Gao G.Q., Wang X.L., Hu S.M., Fan H.B., Fang H.Y. (2022). Effects of Different Exogenous Selenium on Enzyme Activities and Microorganisms in Arseniccontaminated Soil. Appl. Ecol. Env. Res..

[57] Djanaguiraman M., Prasad P.V.V., Seppanen M. (2010). Selenium protects sorghum leaves from oxidative damage under high temperature stress by enhancing antioxidant defense system. Plant Physiol. Biochem..

[58] Mu T.T., Du H.L., Zhang F.Y., Jing X.L., Guo Q., Li Z., Liu Z., Tian G. (2017). Effects of Exogenous Selenium on the Physiological Activity, Grain Selenium Content, Yield and Quality of Foxtail Millet. Sci. Agric. Sin..

[59] Cosh M.H., Jackson T.J., Moran S., Bindlish R. (2008). Temporal persistence and stability of surface soil moisture in a semi-arid watershed. Remote Sens. Environ..

[60] Vanderlinden K., Vereecken H., Hardelauf H., Herbst M., Martínez G., Michael H., Cosh M.H., Pachepsky Y.A. (2012). Temporal stability of soil water contents: A review of data and analyses. Vadose Zone J..

[61] Ahmad I., Cheng Z.H., Meng H.W., Liu T.J., Wang M.Y., Ejaz M., Wasila H. (2013). Effect of Pepper-Garlic Intercropping System on Soil Microbial and Bio-Chemical Properties. Pak. J. Bot..

[62] Frankeberger W.T., Johanson J.B. (1983). Method of Measuring Invertase Activity in Soils. Plant Soil.

[63] Johnson J.L., Temple K.L. (1964). Some Variables Affecting the Measurement of “Catalase Activity” in Soil. Soil Sci. Soc. Am. J..

[64] Buragienė S., Šarauskis E., Romaneckas K., Adamavičienė A., Kriaučiūnienė Z., Avižienytė D., Marozas V., Naujokienė V. (2019). Relationship between CO_2_ Emissions and Soil Properties of Differently Tilled Soils. Sci. Total Environ..

[65] Liu F.S., Zhou Z.B., Hu S.J., Du H.Y., Chen X.L. (2012). Influence of Different Soil Coring Methods on Estimation of Root Distribution Characteristics. Acta Prataculturae Sin..

[66] Costa V.G., Pedreira C.E. (2023). Recent advances in decision trees: An updated survey. Artif. Intell. Rev..

[67] Wang H.F., Jin H.X., Jiang X.J. (2025). Feature Selection for High-Dimensional Varying Coefficient Models via Ordinary Least Squares Projection. Commun. Math. Stat..

